# A Case Report on Tinea Imbricata

**DOI:** 10.7759/cureus.100686

**Published:** 2026-01-03

**Authors:** Haitham Sonbol, Ajlan Alajlani, Omar A Alsuwailem, Yousef L Alsuwailem

**Affiliations:** 1 Dermatology, Prince Mohammed Bin Abdulaziz Hospital, Riyadh Second Health Cluster, Riyadh, SAU; 2 General Practice, College of Medicine, King Faisal University, Al Ahsa, SAU; 3 Dermatology, Riyadh Second Health Cluster, Riyadh, SAU

**Keywords:** dermatophyte, itraconazole, saudi arabia, tinea imbricata, trichophyton concentricum

## Abstract

Tinea imbricata is a rare, chronic, superficial dermatophytosis caused by *Trichophyton concentricum*. The condition is endemic to the Southwest Pacific, Southeast Asia, and parts of Central and South America. We report a case of tinea imbricata in a Yemeni adult who acquired the infection during travel to Yemen. The patient was managed with oral itraconazole (200 mg, pulse therapy) in combination with topical miconazole cream, resulting in complete resolution of lesions. To the best of our knowledge, this is the first reported case of tinea imbricata in Saudi Arabia.

## Introduction

Tinea imbricata (TI) is a rare, chronic superficial mycosis caused by anthropophilic dermatophyte *Trichophyton concentricum* [1]. It manifests as extensive, annular, concentric rings that eventually develop into lamellar, abundant, thick scales, resembling fish scales or overlapping roof tiles. The condition is endemic in the Southwest Pacific, Central and South America, and Southeast Asia, predominantly affecting residents of primitive and isolated environments [2]. Its clinical appearance may resemble other dermatologic conditions, including erythema gyratum repens, tinea indecisiva, and tinea incognito, but these typically lack the characteristic thick lamellar scaling of TI [3,4].

## Case presentation

Table 1 lists the key differences between TI and other dermatological conditions.

**Table 1:** Key clinical differences between tinea imbricata and other scaly concentric dermatoses

Disease	Description
Tinea imbricata	Tinea imbricata, caused by *Trichophyton concentricum*, presents with widespread, annular lesions forming concentric rings of scale and is often associated with itching. The term “imbricata” comes from the Latin *imbrex*, meaning “overlapping roof tile,” reflecting the characteristic appearance of the lesions [3].
Tinea incognito	Tinea incognito is an unusual variant of dermatophyte infection caused by localized immunosuppression due to systemic or topical corticosteroids. The lesions clinically exhibit an absence of well-defined borders, center clearing, and scaling that is often associated with dermatophytosis. The application of corticosteroids may diminish inflammatory indicators, resulting in tinea incognito lesions seeming less erythematous. Trichophyton rubrum is the predominant causal agent, followed by Trichophyton mentagrophytes and Epidermophyton floccosum [4].
Tinea corporis	Tinea corporis, most often due to *T. tonsurans* and less frequently *T. mentagrophytes*, *Microsporum gypseum*, or *M. ferrugineum*, can present—particularly in immunocompromised patients—with hyperpigmented concentric rings that often include areas of hypopigmentation [3].
Erythema gyratum repens	A migratory figurate erythema characterized by multiple concentric rings resembling wood grain, with lesions that can advance up to 1 cm per day and may be accompanied by scaling or itching [3].
Granuloma annulare	A skin-colored to dull pink raised border, often formed by coalescing papules, with a predilection for acral areas and the elbows [3].
Pityriasis versicolor	Also known as tinea versicolor, this condition is caused by the dimorphic, lipid-dependent yeasts *Malassezia furfur* and *Pityrosporum orbiculare*. It is characterized by multiple, well-defined hypo- or hyperpigmented macules or patches with fine scaling, which becomes more apparent when the affected skin is stretched or gently scraped [3].
Annular psoriasis	The scale is silvery and is associated with involvement of the scalp, intergluteal region, and nails [3].

**Figure 1:**
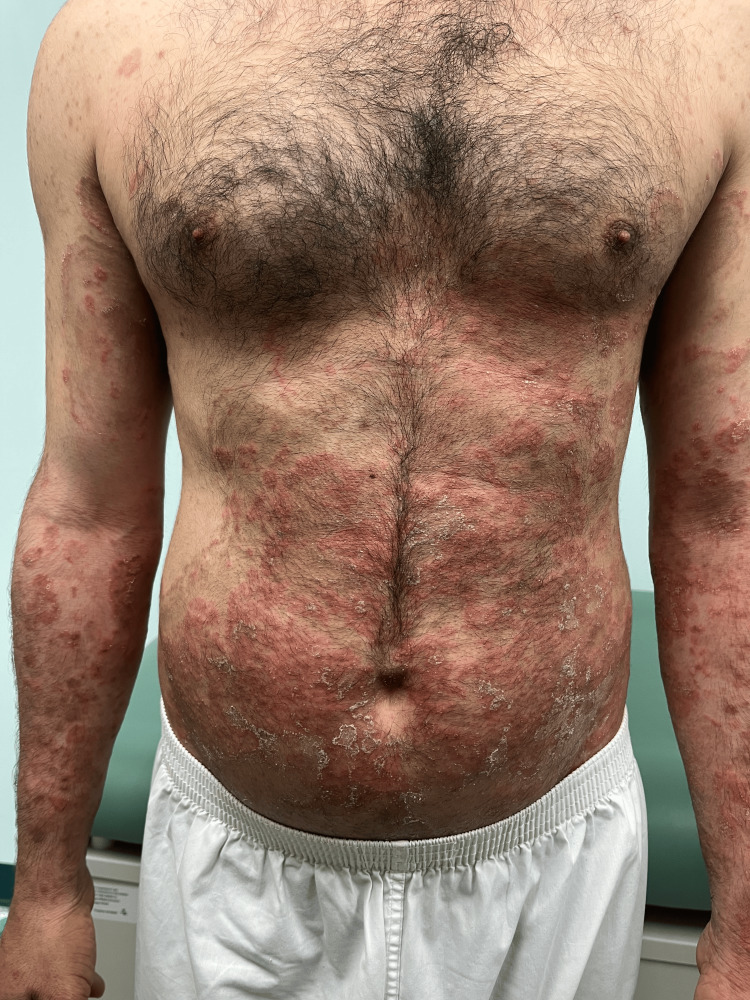
Many well-demarcated confluent erythematous scaly plaques scattered over the trunk and upper limbs

**Figure 2:**
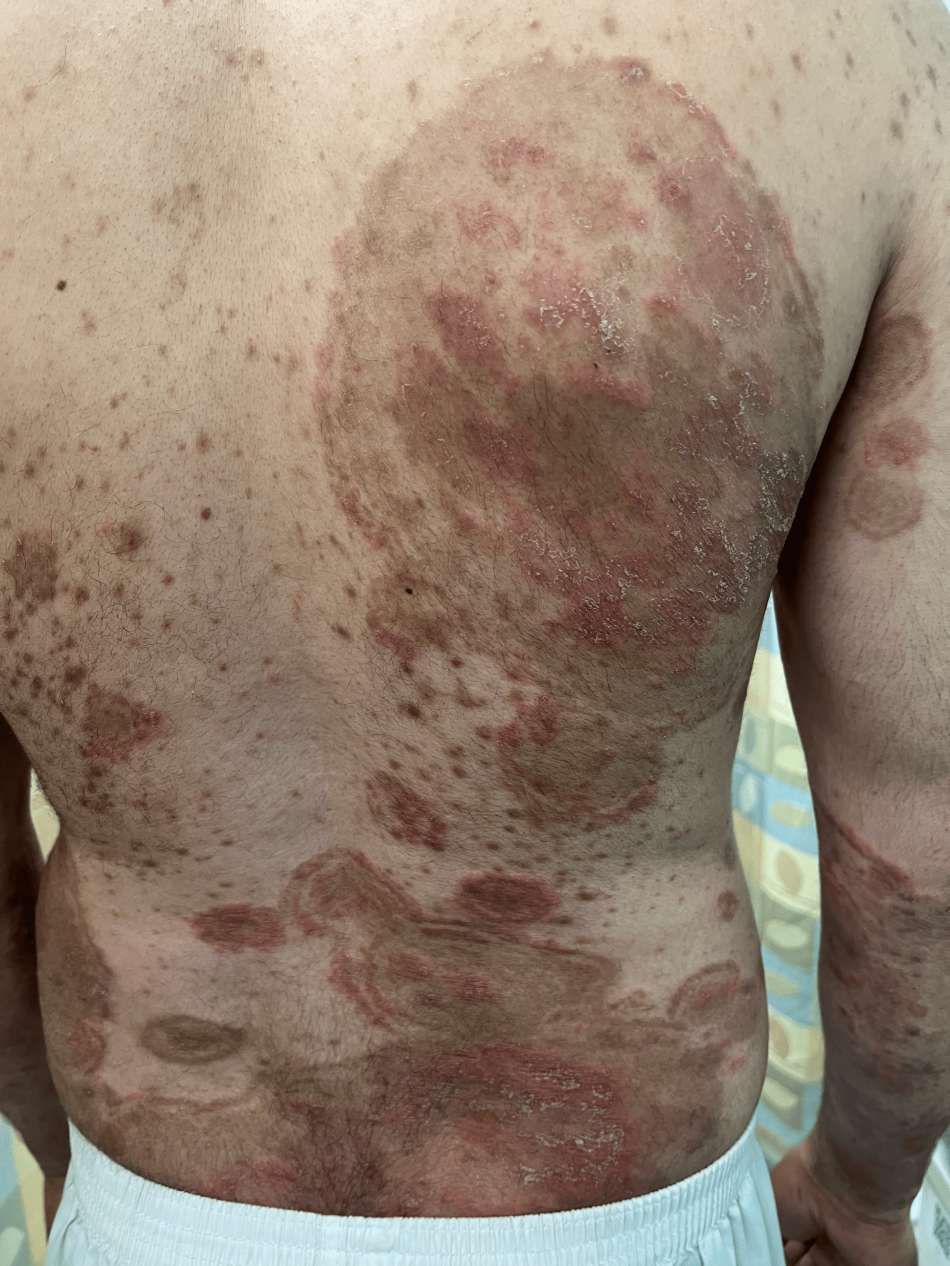
Many polycyclic concentric erythematous plaques with a surrounding brown peri-lesional halo

A 36-year-old man, with no significant medical or surgical history, presented to our dermatology outpatient clinic with a 6-month history of multiple erythematous scaly plaques distributed across his body. Initially, the skin lesions started to appear on his lower and upper limbs, and with time, they progressed to involve his trunk, back, genital area, and face. These skin lesions were associated with severe pruritus, not relieved by an oral antihistamine. There is no personal history, nor a family member or close contact with a similar complaint. He was seen by a primary healthcare physician, and the complaint was labeled as psoriasis. He was given topical betamethasone valerate with calcipotriene once daily for two months. He noticed a 60% improvement with complete resolution of the pruritus. However, once he stopped the topical, he relapsed with greater intensity. Upon examination, we noticed multiple erythematous hypertrophic polycyclic scaly plaques; some of the lesions were surrounded by a perilesional hyperpigmented halo (Figures 1, 2).

**Figure 3:**
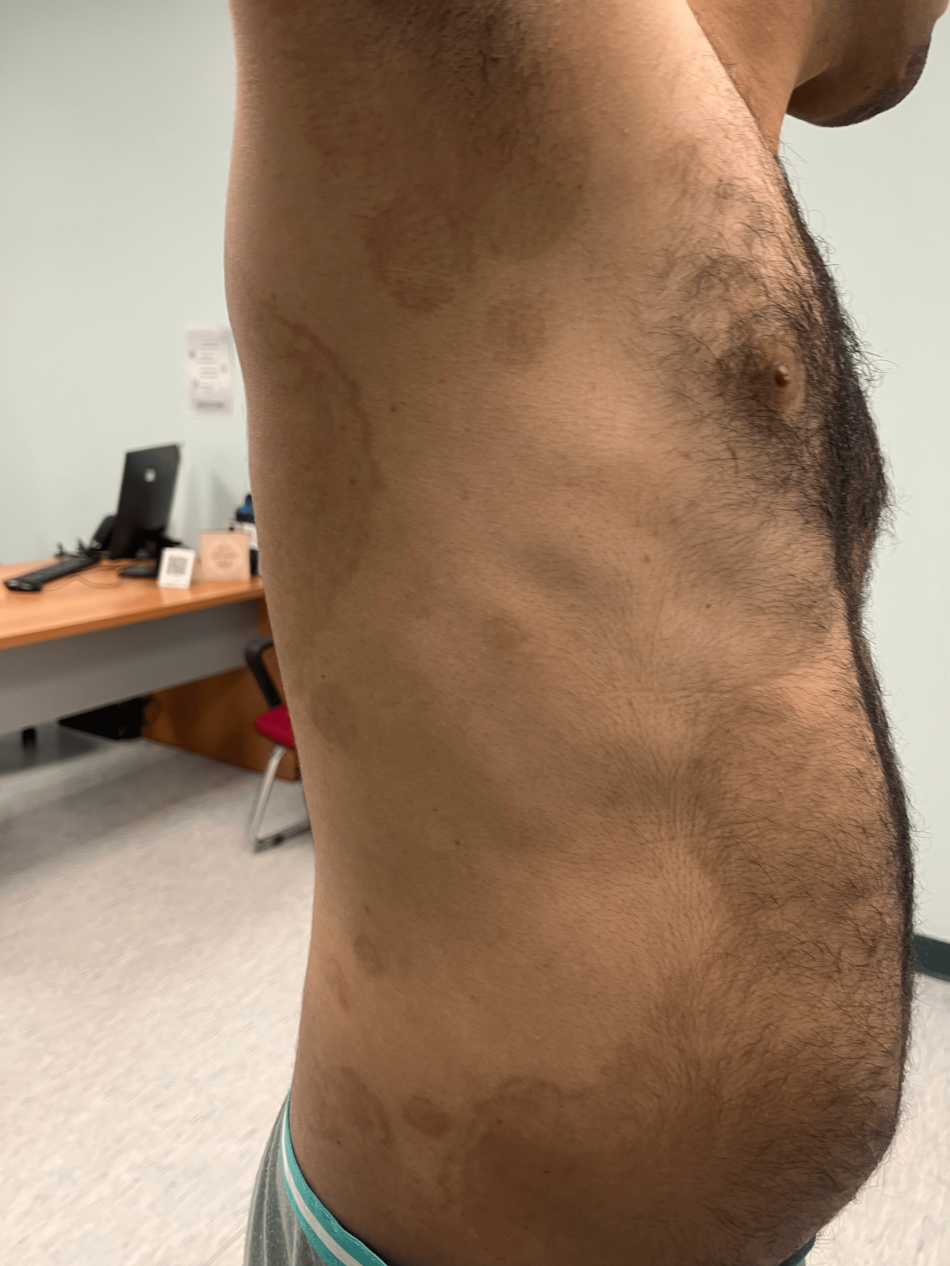
Resolution of the lesions, with post-inflammatory hyperpigmentation

**Figure 4:**
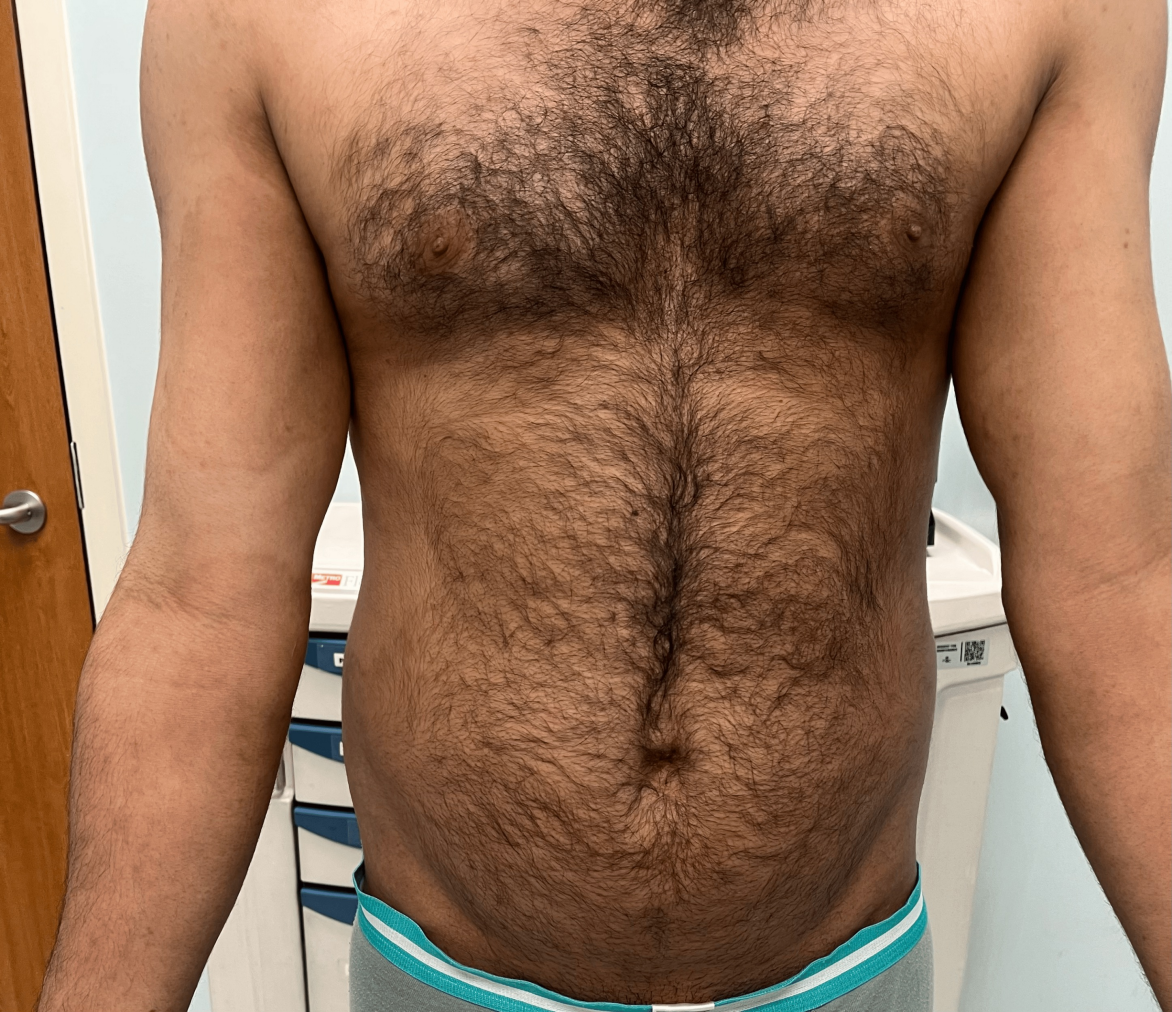
Resolution of the lesions, with post-inflammatory hyperpigmentation (another view)

The diagnosis of TI was made based on his clinical findings. Therefore, given the severity of his presentation, we started him on itraconazole 200 mg in a pulse regimen for 3 months, which is 200 mg daily for the first week of each month, for 3 consecutive months combined with topical miconazole cream applied twice a day over affected areas. After completing the first pulse 1 month later, the patient reported a 70% improvement in pruritus, and we noticed a 60% improvement in skin lesions. Complete resolution was achieved at the three-month follow-up, after the last pulse (Figures 3, 4).

## Discussion

TI is a chronic superficial dermatophytosis caused by *Trichophyton concentricum, *and it is characterized by concentric, polycyclic, scaly plaques that often expand slowly and symmetrically across the skin surface. The condition is endemic to certain tropical and subtropical regions, particularly in the Pacific Islands, Southeast Asia, and parts of Latin America and South America, although sporadic cases may occur globally due to migration and travel [5]. The patient presented with characteristic signs of TI, including widespread, pruritic, erythematous, hypertrophic plaques with peripheral hyperpigmentation. The lack of response to antihistamines suggests an inflammatory rather than allergic etiology. Although laboratory confirmation was not provided, clinical diagnosis is generally reliable in typical or endemic cases [6].

The first documented case of TI was reported by Williams Dampier in 1789. In 1998, a localized case of TI in a 23-year-old British nurse who achieved complete remission after a four-week course of griseofulvin at 1 g per day was also documented [7]. Two pediatric cases of TI have been reported in the literature; both were successfully treated with oral griseofulvin at 10 mg (10 mg/kg/day 4-6 weeks) [1,8].

Diagnosis is usually made clinically based on the skin manifestations of generalized, erythematous, hypertrophic, polycyclic scaly plaques. IT typically affects the face, trunk, and limbs. Involvement of palms, soles, nails, and scalp is uncommon. The disease may present with or without pruritus, and prior use of topical corticosteroids can mask its characteristic features, potentially delaying or complicating diagnosis [2,9]. When necessary, the diagnosis of TI can be confirmed by potassium hydroxide (KOH) examination of skin scrapings taken from the lesion's active edge, which typically reveals short, septate hyphae without arthroconidia and abundant *Trichophyton concentricum* spores. Trichophyton species are identified by the presence of both smooth-walled macroconidia and microconidia, the latter distinguishing them from Epidermophyton and Microsporum. In species lacking conidia, culture features, and clinical context, such as lesion appearance, site, travel history, animal exposure, and occupation, help establish the diagnosis [8].

Treatment of TI is challenging due to its chronic course and tendency to recur. Oral antifungals, such as itraconazole, have shown good efficacy [10]. Additionally, pulse dosing of itraconazole has been shown to be both more effective and safer than continuous administration in the treatment of superficial, deep cutaneous, mucosal, and systemic fungal infections. This approach involves administering high doses of systemic antifungal agents at intermittent intervals to maximize therapeutic efficacy, achieve rapid disease control during acute flares, and minimize adverse effects [11].

The adjunctive use of a topical agent, such as miconazole, may enhance therapeutic efficacy and improve the overall cure rate [3]. In this case, the combination therapy resulted in a 70% reduction in symptoms after one month and complete clinical remission by three months, supporting the effectiveness of dual-antifungal regimens.

## Conclusions

This case contributes to the growing clinical evidence that oral itraconazole combined with topical azoles is a highly effective approach for treating extensive dermatophytic infections such as TI. It also underscores the importance of recognizing characteristic clinical signs to initiate appropriate therapy. Therefore, clinicians should consider TI in patients with widespread concentric scaly plaques and a history of travel to endemic regions, as this awareness is essential for accurate diagnosis and appropriate management.

## References

[1] Veraldi S, Giorgi R, Pontini P, Tadini G, Nazzaro G (2015). Tinea imbricata in an Italian child and review of the literature. Mycopathologia.

[2] Leung AK, Leong KF, Lam JM (2018). Tinea imbricata. J Pediatr.

[3] Leung AK, Leong KF, Lam JM (2019). Tinea imbricata: an overview. Curr Pediatr Rev.

[4] Shony S, Lobo C, Kaimal S (2025). Tinea incognito. Cleve Clin J Med.

[5] Havlickova B, Czaika VA, Friedrich M (2008). Epidemiological trends in skin mycoses worldwide. Mycoses.

[6] Hay R (2013). Superficial fungal infections. Medicine (Abingdon).

[7] Mousavi SAA, Sardoili SS, Shamsadini S (2017). A first case of tinea imbricata from Iran. Jundishapur J Microbiol.

[8] Roslan SR, Abdul Hadi A (2022). A child with unique skin pattern: a case report of Tinea imbricata. Med J Malaysia.

[9] Polunin I (1952). Tinea imbricata in Malaya. Br J Dermatol.

[10] Budimulja U, Kuswadji K, Bramono S (1994). A double-blind, randomized, stratified controlled study of the treatment of tinea imbricata with oral terbinafine or itraconazole. Br J Dermatol.

[11] Del Rosso JQ, Gupta AK (1999). The use of intermittent itraconazole therapy for superficial mycotic infections: a review and update on the 'one week' approach. Int J Dermatol.

